# Perceived cooperative learning climate in physical education and empathy among Chinese upper elementary students: indirect pathways through relatedness need satisfaction and cognitive reappraisal

**DOI:** 10.3389/fpsyg.2026.1852855

**Published:** 2026-08-11

**Authors:** Xianchao Liu, Songbin Wang, Xianlin Xiao

**Affiliations:** 1 School of Physical Education, Chengdu Technological University, Chengdu, China; 2 Faculty of Management, Shinawatra University, Sam Khok, Thailand

**Keywords:** cognitive reappraisal, cooperative learning climate, empathy, physical education, relatedness need satisfaction, self-determination theory, structural equation modeling, upper elementary students

## Abstract

**Purpose:**

This cross-sectional study examined whether perceived cooperative learning climate (PCLC) in physical education (PE) was associated with cognitive empathy (COE) and affective empathy (AFE) among Chinese students in Grades 4–6. Relatedness need satisfaction (RNS) and cognitive reappraisal (COR) were tested as parallel and sequential indirect pathways within a hypothesized structural model.

**Methods:**

A cross-sectional survey was conducted among 2,173 Grade 4–6 students from primary schools in Chengdu, China. Students completed self-report measures of PCLC, RNS, COR, COE, and AFE. Covariance-based structural equation modeling estimated unadjusted direct and specific indirect effects, and bias-corrected bootstrap confidence intervals were calculated using 5,000 resamples. Hierarchical regressions were conducted as supplementary covariate-adjusted analyses.

**Results:**

PCLC was positively associated with COE (β = 0.334) and AFE (β = 0.308). Specific indirect effects through RNS, through COR, and through the sequence RNS→ COR were significant for both empathy outcomes, with standardized estimates ranging from β = 0.024 to β = 0.073. All 95% bootstrapped confidence intervals excluded zero. In supplementary regressions, PCLC, RNS, and COR remained positively associated with both empathy outcomes after demographic variables were entered.

**Conclusion:**

The findings suggest that perceived cooperative learning climate in PE is positively associated with cognitive and affective empathy among Chinese upper elementary students. RNS and COR may represent relational and regulatory pathways linking cooperative PE climates with students' socio-emotional functioning. Longitudinal, multi-informant, and intervention studies are needed to test temporal and causal relations.

## 1 Introduction

Empathy is a core socio-emotional competence that supports prosocial behavior, peer acceptance, and constructive conflict management during late childhood (Morizio et al., 2024). In upper elementary school, students' daily learning increasingly depends on collaboration, perspective taking, and emotionally attuned interaction with classmates (Chokri and Jarraya, 2024). Empathy during this period can therefore be understood not only as an individual disposition, but also as a context-sensitive capability shaped by the relational and emotional demands of school life (Pan et al., 2025). Growing scholarly attention to emotional intelligence and socio-emotional capacities in education further underscores the need to examine how everyday school environments are associated with students' social and emotional development (Solih et al., 2024). A key question is which instructional features are linked with students' empathy, and through what psychological processes (Swan, 2021).

Physical education (PE) provides a particularly relevant context for addressing this question (Malinauskas and Malinauskiene, 2021). PE lessons often require students to work with peers, coordinate actions toward shared goals, and manage visible emotional experiences such as excitement, frustration, pride, and embarrassment. These features make PE a natural setting for social learning, while also making peer evaluation, exclusion, and emotional safety especially salient when instructional structures are poorly aligned (García-González et al., 2023). This may be particularly consequential in Chinese upper elementary PE, where students often interact with the same classmates across repeated, highly visible group activities. Cooperative learning approaches have been widely advocated in such contexts because they emphasize positive interdependence, promotive interaction, group processing, individual accountability, and social skills, thereby helping group activity become genuine cooperation rather than simple co-presence (Boke et al., 2025). When these elements are experienced as stable features of PE lessons, the classroom climate may provide repeated opportunities for students to notice others' feelings, coordinate with peers, negotiate disagreement, and respond supportively.

Despite this promise, several limitations remain in the PE and school-based social–emotional learning literature. Evidence linking cooperative learning to social outcomes is often framed in terms of program effectiveness or overall associations, whereas the psychological processes through which cooperative classroom structures may relate to empathy have received less direct empirical attention (Boke et al., 2025). Empathy is also commonly treated as a broad outcome, although it includes at least two related but distinct components: cognitive empathy, which concerns understanding others' feelings and perspectives, and affective empathy, which concerns emotional resonance and empathic concern (Pla-Pla et al., 2024). Treating empathy as a single undifferentiated construct may obscure how instructional climates relate to perspective-oriented understanding and affective responsiveness during late childhood (Wang et al., 2024). In addition, evidence remains limited for Chinese upper elementary PE as a meaningful context in its own right, rather than merely as a sampling location.

A mechanism-oriented perspective suggests that cooperative learning climates may be associated with empathy through students' relational experiences and emotion-related self-regulation (Rivera-Pérez et al., 2021). Cooperative activities reorganize peer interaction by requiring students to communicate, coordinate, and support one another's success (Rivera-Pérez et al., 2020). When these expectations are enacted in a safe and inclusive classroom climate, students may experience stronger social connection and belonging. Such relational security may reduce self-protective vigilance and make students more open to peers' feelings and viewpoints (Meza et al., 2025). PE also exposes students to emotionally charged social situations, including public mistakes, competitive arousal, peer feedback, and coordination difficulties. In these moments, students who can reinterpret stressful events in a constructive way may be better able to remain engaged with others' experiences rather than becoming overwhelmed by self-focused distress (Törmänen et al., 2025).

The present study focuses on two candidate processes that may help explain these links: relatedness need satisfaction (RNS), referring to students' felt acceptance, connection, and interpersonal comfort in PE (Li et al., 2024), and cognitive reappraisal (COR), referring to the reinterpretation of a situation in order to alter its emotional impact (Xu et al., 2023). These processes can be integrated in a sequential pathway. A more cooperative learning climate may strengthen students' sense of belonging and interpersonal security; this relational experience may reduce threat appraisals in challenging peer situations and make cognitive reappraisal more feasible; more adaptive reappraisal may then help students remain open to others' experiences, thereby relating to both cognitive and affective empathy (Muntamah and Pratama, 2025; Xu et al., 2023). This proposed ordering provides a coherent framework for examining how cooperative PE climates may be linked with empathy through relational and regulatory processes, while remaining consistent with the cross-sectional nature of the data.

The present study also models cognitive empathy and affective empathy as distinct but correlated outcomes within the same structural framework (Kusumaningsih and Sun, 2025; Rivera-Pérez et al., 2021). This distinction allows the analysis to examine whether cooperative learning climate, relatedness need satisfaction, and cognitive reappraisal show similar or different patterns of association with the two empathy dimensions (Simon and Nader-Grosbois, 2025). Such differentiation is especially relevant in Grades 4–6, when social-cognitive sophistication develops rapidly and affective responsiveness remains sensitive to peer climate and emotion-regulation demands (Kusumaningsih and Sun, 2025).

This study extends prior work by examining Chinese upper elementary PE as a socially consequential context for students' socio-emotional functioning, distinguishing cognitive empathy from affective empathy, and integrating cooperative learning climate with relatedness need satisfaction and cognitive reappraisal in one model. Accordingly, the study examines whether perceived cooperative learning climate in PE is associated with cognitive and affective empathy, and whether RNS and COR serve as parallel and sequential indirect pathways within the hypothesized structural model.

## 2 Literature review and hypotheses

### 2.1 Empathy as a dual-dimensional outcome in upper elementary students

Empathy is widely regarded as a core socio-emotional competence that supports prosocial behavior, peer adjustment, and constructive social participation during late childhood. Contemporary conceptualizations commonly distinguish cognitive empathy (COE) from affective empathy (AFE). COE refers to the capacity to understand another person's emotional state and perspective, whereas AFE refers to emotional resonance or empathic concern in response to another person's experience (Ayache et al., 2024; Vallette d'Osia and Meier, 2024). Although the two components are related, they represent distinguishable functions and may show different antecedents and correlates (Goodhew and Edwards, 2021; Israelashvili and Karniol, 2018).

This distinction is particularly relevant in upper elementary school. During Grades 4–6, children show notable development in perspective taking, emotion knowledge, and peer-oriented social behavior. Such developmental changes may be especially relevant for COE, which depends on interpreting others' perspectives and emotional cues. By contrast, AFE may remain more closely tied to interpersonal signals such as inclusion, warmth, and relational safety in daily school life (Chen et al., 2025). As peer interactions become more complex in late childhood, children increasingly need to interpret classmates' intentions, respond to emotional cues, and regulate their own reactions to social evaluation (Chen et al., 2025). These characteristics suggest that COE and AFE may not relate identically to classroom environments that organize peer interaction (Theophilou et al., 2024).

For this reason, treating empathy as a dual-dimensional outcome can increase theoretical precision when examining school-based correlates. In PE, where peer interaction is frequent and emotions are often expressed in real time during movement-based tasks, a cooperative instructional environment may be associated with students' perspective-oriented understanding and emotional responsiveness in partly overlapping but non-identical ways. Modeling COE and AFE as distinct but correlated outcomes provides a more differentiated basis for examining how cooperative learning climate and related psychological variables are associated with empathy in upper elementary students.

### 2.2 Cooperative learning climate in relation to empathy

A perceived cooperative learning climate in physical education (PCLC) refers to students' perceptions that PE lessons are organized around cooperative learning principles rather than primarily competitive or individually oriented practices (Fernández-Rio et al., 2017). Core elements typically include positive interdependence, promotive interaction, individual accountability, group processing, and social skills. Together, these features structure peer interaction around shared goals, mutual support, communication, and constructive conflict handling.

PE provides a distinctive setting for examining how such a climate may be associated with empathy (Costa et al., 2025; Rivera-Pérez et al., 2020). Lessons often involve public performance, peer observation, immediate feedback, and emotionally salient outcomes such as winning, failing, being selected, or being evaluated by classmates. These conditions create repeated opportunities to notice others' emotional states, interpret peers' responses, and regulate one's own interpersonal reactions during collaboration. Cooperative learning climates can increase meaningful peer contact and establish norms that reward supportive behavior, which may be relevant to both perspective taking and emotional responsiveness in group activity (Železnik Mežan et al., 2025).

Theoretically, PCLC may be positively associated with both components of empathy in late childhood (Boke et al., 2025). For COE, cooperative task demands often require students to understand classmates' intentions, constraints, and feelings in order to coordinate effectively. For AFE, cooperative norms may make peers' experiences more salient and socially meaningful, thereby relating to greater empathic concern and emotional attunement. Accordingly, the following hypotheses are proposed:

H1a: PCLC is positively associated with COE.
H1b: PCLC is positively associated with AFE.

### 2.3 Relatedness need satisfaction as a relational indirect pathway

Self-determination theory proposes that social contexts become psychologically consequential in part through the satisfaction of basic psychological needs (de Bruijn et al., 2022). Relatedness need satisfaction in physical education (RNS) refers to students' felt belonging, acceptance, and interpersonal connection during PE lessons (de Bruijn et al., 2022; Li et al., 2024). High RNS indicates comfort in peer interaction, a sense of being valued, and reduced concern about rejection or exclusion in the activity setting (Li et al., 2024; de Bruijn and Grimminger-Seidensticker, 2026). In late childhood, peer belonging is a particularly salient resource because it shapes emotional security, engagement, and willingness to participate socially in class-based tasks (Gempp and González-Carrasco, 2021; Chen and Hypnar, 2015).

A cooperative learning climate provides a plausible basis for stronger RNS because it structures peer interaction in ways that may increase supportive contact and reduce social threat (Kipp and Bolter, 2024). Positive interdependence encourages students to view classmates as partners rather than obstacles, while promotive interaction fosters helping and encouragement. Individual accountability may reduce tensions related to uneven effort, and group processing creates space for recognition and shared reflection. Social skills elements further support respectful communication and constructive responses to disagreement (Gorozidis et al., 2020). Taken together, these features may increase perceived inclusion and interpersonal comfort in PE (Ghaith et al., 2007; Gorozidis et al., 2020; Kipp and Bolter, 2024).

RNS is also theoretically relevant to empathy because relational security may shape attention, motivation, and interpersonal openness (Rivera-Pérez et al., 2021). When belonging and acceptance are salient, students may be less preoccupied with self-protection and more open to classmates' emotional cues and perspectives (Hortigüela Alcalá et al., 2019). In this way, stronger RNS may be associated with COE through greater willingness to understand others' viewpoints (García-González et al., 2023), and with AFE through greater empathic concern and emotional resonance in a supportive peer context (Fousiani et al., 2016; Lloyd and Smith, 2022). On this basis, the following hypotheses are proposed:

H2a: PCLC has a significant specific indirect effect on COE through RNS.
H2b: PCLC has a significant specific indirect effect on AFE through RNS.

### 2.4 Cognitive reappraisal as a regulatory indirect pathway

Cognitive reappraisal (COR) refers to a regulatory strategy in which individuals reinterpret an emotion-eliciting situation in order to alter its emotional impact (Gross, 1998). Within process-oriented accounts of emotion regulation, reappraisal is categorized as an antecedent-focused strategy because it changes the meaning of a situation before emotional responses fully unfold (Gross, 1998, 2015). In upper elementary school, reappraisal becomes increasingly feasible as children's cognitive flexibility, perspective taking, and reflective capacities develop (Denny and Ochsner, 2014). Compared with response-focused strategies, reappraisal is generally associated with better socio-emotional adjustment because it can reduce negative affect while maintaining engagement with the social context (John and Gross, 2004).

A perceived cooperative learning climate in PE may also be relevant to COR because it shapes both the frequency of emotionally challenging interactions and the interpretive norms applied to those interactions (Zhou et al., 2025). Cooperative tasks often involve disagreement, uneven contribution, and public mistakes, all of which may elicit frustration, embarrassment, or anger (Zhou et al., 2025). When the lesson climate emphasizes positive interdependence and promotive interaction, such setbacks may be construed more readily as shared problems to be addressed rather than as personal failures. Group processing further invites students to reflect on what happened and how to improve, which is compatible with the cognitive reframing processes that define reappraisal. In this sense, cooperative climates may provide repeated conditions for constructive meaning-making in emotionally charged peer situations (Železnik Mežan et al., 2025).

COR is further relevant to empathy because empathy requires attention to others' emotional cues and a reflective stance toward interpersonal events (Morawetz et al., 2022). Strong negative affect and self-focused distress can narrow attention and reduce openness to others, which may undermine both perspective taking and empathic concern (Lockwood et al., 2014). Reappraisal may help students reinterpret stressful interpersonal situations in ways that allow them to remain engaged with peers' experiences. Thus, COR may be positively associated with COE by supporting perspective integration and emotion understanding, and with AFE by sustaining empathic concern without escalating personal distress (Thompson et al., 2022). On this basis, the following hypotheses are proposed:

H3a: PCLC has a significant specific indirect effect on COE through COR.
H3b: PCLC has a significant specific indirect effect on AFE through COR.

### 2.5 A sequential pathway involving relatedness need satisfaction and cognitive reappraisal

Beyond these two specific indirect pathways, the study considers a sequential pathway linking PCLC, RNS, COR, and empathy. In this sequence, RNS is positioned upstream of COR. The rationale is that relational security may function as a psychological resource that supports more adaptive regulation in socially demanding activities (Deci and Ryan, 2014; Vanhee et al., 2016). When students feel accepted and connected in PE, peer interactions may be less likely to be appraised as threatening, and cognitive reframing may become more feasible during emotionally arousing events such as conflict, evaluation, or coordination failure (Krafft et al., 2019).

Within cooperative PE lessons, stronger RNS may contribute to COR by changing the meaning context in which emotions arise (Wang et al., 2024). High relatedness may increase trust in peers' intentions, reduce anticipatory worry about rejection, and normalize temporary setbacks as part of collective learning. These conditions may lessen defensive responding and encourage reflective processing, which is central to reappraisal. Accordingly, a cooperative climate may first be associated with students' sense of connection and belonging (RNS) (Riley, 2016), which may in turn be associated with greater use of cognitive reappraisal (COR) in peer situations. This proposed ordering provides a coherent basis for examining how relational and regulatory processes may be jointly linked with empathy, without implying that it is the only plausible sequence.

This sequential process is expected to be relevant to both empathy components. For COE, relational security and reappraisal may jointly relate to perspective taking by reducing self-focused threat and enabling greater attention to others' appraisals and emotions. For AFE, the same sequence may be associated with empathic concern by preserving emotional openness while reducing the likelihood that interpersonal stress escalates into self-focused distress (Guo et al., 2022; León et al., 2023). Therefore, the following hypotheses are proposed:

H4a: PCLC has a significant sequential indirect effect on COE through RNS followed by COR.
H4b: PCLC has a significant sequential indirect effect on AFE through RNS followed by COR.

### 2.6 Summary of hypotheses and proposed model

**Figure 1:**
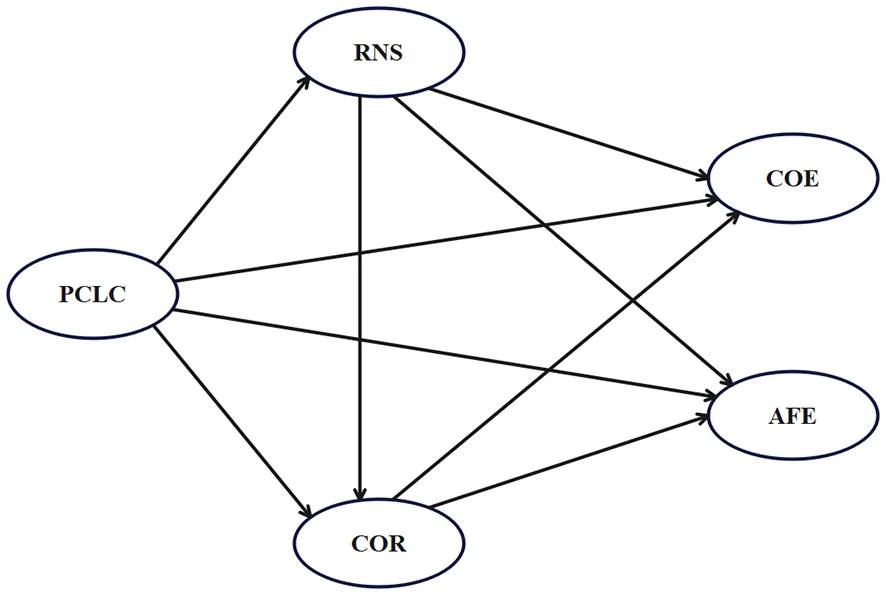
Proposed structural model linking cooperative learning climate to cognitive and affective empathy in physical education. PCLC, perceived cooperative learning climate in physical education; RNS, relatedness need satisfaction in physical education; COR, cognitive reappraisal; COE, cognitive empathy; AFE, affective empathy. Solid arrows represent hypothesized associations. Indirect effects include paths through RNS, through COR, and through the sequence RNS→ COR.

In summary, the present study proposes that perceived cooperative learning climate in physical education (PCLC) is positively associated with cognitive empathy (COE) and affective empathy (AFE) (H1a–H1b). Two specific indirect effects are further specified: one through relatedness need satisfaction (RNS) (H2a–H2b) and one through cognitive reappraisal (COR) (H3a–H3b). In addition, the model includes a sequential indirect effect in which PCLC is associated with empathy through RNS followed by COR (H4a–H4b). These direct paths and indirect effects are integrated in the proposed structural model shown in Figure 1.

## 3 Materials and methods

### 3.1 Participants and data collection

This study used a cross-sectional survey design. Data were collected from upper elementary school students in Grades 4–6 between December 1 and December 30, 2025, in Chengdu, Sichuan Province, China. Schools were recruited on the basis of institutional access and willingness to participate. Within the participating schools, 125 Grade 4–6 classes were included, and approximately 20 students were randomly selected from each class. The sampling procedure therefore involved random student selection within participating classes, with a convenience component at the school-recruitment stage. The sample should be understood as a large multi-school sample from Chengdu rather than a probability-representative sample of all Chinese primary school students.

Ethical approval was obtained from the Ethics Committee of the School of Physical Education, Chengdu Technological University (Approval No. CDTU2025TY015). Because all participants were minors, written informed consent was obtained from parents or legal guardians, and written assent was obtained from students. Participation was voluntary and anonymous. Students were informed that the survey was for research purposes only, that there were no right or wrong answers, and that they could decline participation or withdraw without penalty.

Questionnaires were administered in paper-and-pencil format during regular school hours in classrooms or designated school activity rooms. Trained research assistants introduced the study, explained the response procedures, and answered procedural questions. Students completed the questionnaires independently, and teachers were asked not to intervene in students' responses. Completion required approximately 15–20 min.

A total of 2,500 questionnaires were distributed, and 2,390 were returned, yielding a response rate of 95.60%. Returned questionnaires were screened using prespecified data-quality criteria. Questionnaires were excluded if they had more than 10% missing item responses, showed invariant or long-string responding on at least 80% of the items, or contained clear logical inconsistencies suggestive of inattentive responding. The 80% long-string criterion was used as a conservative screening rule to identify highly invariant response patterns. Of the 2,390 returned questionnaires, 11 were excluded because of excessive missing data, 96 because of invariant responding, and 110 because of logically inconsistent responses. The final analytic sample included 2,173 students, corresponding to a valid response rate of 90.92% among returned questionnaires and 86.92% among all distributed questionnaires.

The final sample comprised 1,109 boys (51.05%) and 1,064 girls (48.95%). By grade, 706 students were in Grade 4 (32.49%), 748 in Grade 5 (34.43%), and 719 in Grade 6 (33.08%). Participants ranged in age from 9 to 13 years, with a mean age of 11.02 years (SD = 0.84). Additional demographic characteristics are reported in Table 1.

**Table 1:** Demographic characteristics of the sample (*N* = 2,173).

Variable	Category	*n*	%
Gender	Boys	1,109	51.05
	Girls	1,064	48.95
Grade	Grade 4	706	32.49
	Grade 5	748	34.43
	Grade 6	719	33.08
Residential area	Urban	1,284	59.08
	Town	543	24.99
	Rural	346	15.93
Only-child status	Yes	1,027	47.26
	No	1,146	52.74
Parental education (Highest level)	Associate degree or below	601	27.66
	Bachelor's degree	1,028	47.31
	Master's degree or above	544	25.03

After case-level screening, the retained dataset contained only low levels of residual item-level missingness. Missing data in the confirmatory factor analysis and structural equation modeling were handled using full information maximum likelihood estimation in AMOS. The final sample size of 2,173 was considered adequate for the planned CFA, covariance-based SEM, and bootstrap estimation of direct and indirect effects.

### 3.2 Measurement tools

All constructs were assessed using Chinese self-report questionnaires. All scales used a 5-point Likert-type response format, and higher scores indicated higher levels of the corresponding construct. Reverse-worded items were recoded before analysis. For instruments with an available Chinese version, the Chinese version was reviewed for age appropriateness and linguistic clarity. For instruments requiring PE contextualization, translation, back-translation, expert review, and pilot testing were conducted before the main survey. The pilot test involved 36 upper elementary students from two non-participating schools, and minor wording changes were made to improve comprehensibility and contextual fit for PE classes.

#### 3.2.1 Perceived cooperative learning climate in physical education

Perceived cooperative learning climate in physical education (PCLC) was assessed using a PE-contextualized version of the Cooperative Learning Questionnaire (CLQ; Fernández-Rio et al., 2017). The instrument includes 20 items covering five dimensions: social skills, group processing, positive interdependence, promotive interaction, and individual accountability. Item wording was adapted to refer specifically to PE lessons while preserving the original conceptual content. In the measurement model, PCLC was specified as a second-order latent construct indicated by the five first-order dimensions. Cronbach's α values for the five subscales ranged from 0.801 to 0.855.

#### 3.2.2 Relatedness need satisfaction

Relatedness need satisfaction in physical education (RNS) was measured using the four-item Relatedness subscale adapted from the Basic Psychological Needs in Exercise Scale (BPNES; Vlachopoulos and Michailidou, 2006). Because the original BPNES was developed for exercise settings, item wording was adapted to refer to PE classes and classmates. The adapted scale reflected perceived belonging, acceptance, and interpersonal comfort in PE. Cronbach's α was 0.847.

#### 3.2.3 Cognitive reappraisal

Cognitive reappraisal (COR) was measured using the six-item Cognitive Reappraisal subscale of the Emotion Regulation Questionnaire for Children and Adolescents (ERQ-CA; Gullone and Taffe, 2012). The Chinese version was reviewed for linguistic clarity and age appropriateness before administration. Higher scores indicated greater habitual use of cognitive reappraisal strategies. Cronbach's α was 0.874.

#### 3.2.4 Empathy

Empathy was assessed using the Chinese adolescent version of the Basic Empathy Scale (BES; Li et al., 2011). The scale contains 20 items and includes two dimensions: cognitive empathy (COE; nine items) and affective empathy (AFE; 11 items). The Chinese version was considered consistent with the dual-component view of empathy adopted in this study. Item wording was checked during pilot testing to ensure comprehensibility for upper elementary students. In the structural model, COE and AFE were treated as two correlated first-order latent variables. Cronbach's α values were 0.913 for COE and 0.931 for AFE.

### 3.3 Data analysis

All analyses were conducted using IBM SPSS Statistics 26.0 and AMOS 26.0. Preliminary screening was conducted before model testing. Descriptive statistics were computed for PCLC, RNS, COR, COE, and AFE. Because students were sampled within classes, the nested structure of the data was evaluated by calculating ICC(1), ICC(2), and design effects for the core study variables at the class level. The obtained values were low overall (ICC(1) values = 0.01–0.04, ICC(2) values = 0.15–0.42, and design effects = 1.17–1.66), indicating limited clustering influence on the focal constructs. Single-level CFA and SEM were therefore used, while the clustered sampling structure was acknowledged as a methodological consideration.

Potential common method variance was examined using exploratory and confirmatory approaches (Podsakoff and Organ, 1986; Podsakoff et al., 2003). Harman's single-factor test was used as a descriptive diagnostic. The hypothesized measurement model was then compared with a constrained single-factor CFA model. An unmeasured latent method construct was also specified to load on all observed indicators to assess whether the inclusion of a common method factor materially improved model fit. Because all focal variables were derived from student self-report, common method variance could not be ruled out entirely.

Internal consistency reliability was assessed using Cronbach's α. Construct reliability and convergent validity were evaluated using composite reliability (CR), average variance extracted (AVE), and standardized factor loadings. Convergent validity was considered adequate when CR values exceeded 0.70, AVE values exceeded 0.50, and standardized factor loadings exceeded 0.60 (Fornell and Larcker, 1981). Following the two-step approach recommended by Anderson and Gerbing (1988), CFA was conducted to test the hypothesized measurement model including PCLC, RNS, COR, COE, and AFE. Model fit was evaluated using χ^2^/df, CFI, TLI, SRMR, and RMSEA with its 90% confidence interval (Hu and Bentler, 1999). Discriminant validity was assessed by comparing the hypothesized five-factor model with alternative measurement models and by calculating the heterotrait–monotrait ratio of correlations (HTMT). HTMT values below 0.85 were interpreted as evidence of discriminant validity under a conservative criterion, and values below 0.90 were considered acceptable under a more lenient criterion (Henseler et al., 2015).

Pearson correlations were computed to examine bivariate associations among the study variables. Hierarchical multiple regressions were conducted as supplementary covariate-adjusted analyses. Demographic variables, including gender, grade, residential area, only-child status, and parental education, were entered in the first step; PCLC was entered in the second step; and RNS and COR were entered in the final step.

The hypothesized structural model was tested using covariance-based SEM in AMOS 26.0. The model specified direct paths from PCLC to COE and AFE, specific indirect pathways through RNS and COR, and a sequential indirect pathway through RNS followed by COR. COE and AFE were modeled as correlated endogenous variables. The SEM did not include demographic covariates; therefore, all SEM direct and indirect effects should be interpreted as unadjusted latent-variable estimates. Standardized path coefficients were reported for all structural paths.

Indirect effects were examined using a bias-corrected bootstrap procedure with 5,000 resamples. Indirect effects were considered statistically significant when the 95% confidence interval did not include zero. The bootstrap procedure enabled simultaneous estimation of specific indirect effects through RNS, through COR, and through the sequence RNS→ COR. Given the cross-sectional design, these analyses were interpreted as tests of indirect effects within a hypothesized statistical process model and not as evidence of causal mediation.

## 4 Results

### 4.1 Common method bias test

Common method variance (CMV) was examined using Harman's single-factor test, CFA model comparisons, and an unmeasured latent method construct (ULMC) model. First, Harman's single-factor test was conducted through unrotated exploratory factor analysis (EFA) including all measurement items. The results extracted nine factors with eigenvalues > 1, accounting for a cumulative variance of 63.47%. The first unrotated factor explained 27.73% of the total variance. As a descriptive diagnostic, this result indicated that no single factor dominated the covariance among the items. Second, confirmatory factor analyses (CFA) were conducted to compare the hypothesized measurement model with more constrained factor structures. As shown in Table 2, the one-factor model demonstrated poor fit (χ^2^/df = 28.895, CFI = 0.598, TLI = 0.572, SRMR = 0.107, RMSEA = 0.113), indicating that the data could not be adequately represented by a single latent construct. To further examine whether the focal constructs were empirically distinguishable, a series of increasingly differentiated alternative measurement models was tested by progressively separating the combined constructs. Model fit improved steadily as additional factors were specified. The hypothesized five-factor model, in which PCLC, RNS, COR, COE, and AFE were modeled as distinct latent variables, showed excellent fit (χ^2^/df = 2.600, CFI = 0.977, TLI = 0.975, SRMR = 0.027, RMSEA = 0.027), substantially outperforming the more constrained one-, two-, three-, and four-factor models.

**Table 2:** Comparison of alternative measurement models and common method bias test (*N* = 2,173).

Model	χ^2^/df	CFI	TLI	SRMR	RMSEA [90% CI]
One-factor	28.895	0.598	0.572	0.107	0.113 [0.101, 0.119]
Two-factor	19.376	0.735	0.718	0.089	0.092 [0.082, 0.096]
Three-factor	12.391	0.837	0.825	0.076	0.072 [0.065, 0.076]
Four-factor	7.443	0.908	0.901	0.060	0.054 [0.049, 0.057]
Five-factor	2.600	0.977	0.975	0.027	0.027 [0.025, 0.029]
ULMC-factor	2.605	0.977	0.975	0.027	0.027 [0.025, 0.029]

One-factor model: all items of PCLC, RNS, COR, COE, and AFE loaded on a single latent factor. Two-factor model: all PCLC, RNS, COR, and COE items loaded on one factor, and all AFE items loaded on a second factor. Three-factor model: all PCLC, RNS, and COR items loaded on one factor, all COE items loaded on a second factor, and all AFE items loaded on a third factor. Four-factor model: all PCLC and RNS items loaded on one factor, whereas COR, COE, and AFE were specified as three separate factors. Five-factor model: PCLC, RNS, COR, COE, and AFE were specified as five distinct latent factors. ULMC-factor model: the hypothesized five-factor model with an additional unmeasured latent method construct loading on all observed indicators. ULMC, unmeasured latent method construct; CFI, comparative fit index; TLI, Tucker–Lewis index; SRMR, standardized root mean square residual; RMSEA, root mean square error of approximation; CI, confidence interval.

To further probe potential method-related bias, an unmeasured latent method construct (ULMC) model was estimated by specifying a common latent factor loading on all observed indicators. As presented in Table 2, the ULMC-factor model did not yield a meaningful improvement in fit relative to the hypothesized five-factor model. The global fit indices were virtually identical (χ^2^/df = 2.605, CFI = 0.977, TLI = 0.975, SRMR = 0.027, RMSEA = 0.027), suggesting that inclusion of a common latent factor did not materially alter model performance. Taken together, the CFA comparisons and ULMC results indicated that CMV was unlikely to fully account for the observed associations, although it could not be ruled out because all focal constructs were derived from student self-report.

### 4.2 Descriptive statistics and measurement reliability

Descriptive statistics and reliability indices are presented in Table 3. Internal consistency reliability was satisfactory to excellent, with Cronbach's α values ranging from 0.801 to 0.931. Standardized factor loadings, CR values, and AVE values met the recommended criteria, supporting indicator reliability, construct reliability, and convergent validity for all constructs.

**Table 3:** Descriptive statistics and measurement reliability of study variables (*N* = 2,173).

Variable	Mean	SD	α	Factor loading	CR	AVE
PCLC	3.61	0.70	0.801–0.855	0.726,0.767	0.863	0.557
RNS	3.66	0.97	0.847	0.751,0.770	0.848	0.582
COR	3.66	0.89	0.874	0.713,0.772	0.879	0.547
COE	3.73	0.86	0.913	0.714,0.759	0.914	0.540
AFE	3.60	0.90	0.931	0.702,0.764	0.932	0.554

PCLC, perceived cooperative learning climate in physical education; RNS, relatedness need satisfaction in physical education; COR, cognitive reappraisal; COE, cognitive empathy; AFE, affective empathy. α, Cronbach's alpha; CR, composite reliability; AVE, average variance extracted. Factor loading values represent the range of standardized loadings for each construct.

### 4.3 Correlation analysis and discriminant validity

Pearson correlation analyses were conducted to examine the bivariate associations among PCLC, RNS, COR, COE, and AFE. As shown in Table 4, all correlations were positive and statistically significant (*p* < 0.001). PCLC was moderately correlated with RNS (*r* = 0.287), COR (*r* = 0.311), COE (*r* = 0.410), and AFE (*r* = 0.402). RNS was positively associated with COR (*r* = 0.328), COE (*r* = 0.327), and AFE (*r* = 0.340). COR was positively related to both COE (*r* = 0.385) and AFE (*r* = 0.403). The strongest correlation was observed between COE and AFE (*r* = 0.519), indicating that the two empathy dimensions were related but empirically distinguishable.

**Table 4:** Correlations and discriminant validity among key variables (*N* = 2,173).

Variable	PCLC	RNS	COR	COE	AFE
PCLC	0.746				
RNS	0.287^***^	0.763			
COR	0.311^***^	0.328^***^	0.740		
COE	0.410^***^	0.327^***^	0.385^***^	0.735	
AFE	0.402^***^	0.340^***^	0.403^***^	0.519^***^	0.744

PCLC, perceived cooperative learning climate in physical education; RNS, relatedness need satisfaction in physical education; COR, cognitive reappraisal; COE, cognitive empathy; AFE, affective empathy. Values on the diagonal represent the square roots of the average variance extracted (AVE). ^***^p < 0.001.

Discriminant validity was first assessed using the Fornell–Larcker criterion. As shown in Table 4, the square root of AVE for each construct exceeded its correlations with the other constructs, providing initial evidence of discriminant validity.

Discriminant validity was further evaluated using the heterotrait–monotrait ratio of correlations (HTMT). As shown in Table 5, all HTMT values ranged from 0.344 to 0.562, which were below the conservative threshold of 0.85 and the more lenient threshold of 0.90. The highest HTMT value was observed between COE and AFE (0.562), indicating acceptable empirical distinctiveness. Together, the Fornell–Larcker and HTMT results supported adequate discriminant validity among the five constructs.

**Table 5:** Heterotrait–monotrait ratio (HTMT) results for discriminant validity among the study constructs (*N* = 2,173).

Variable	PCLC	RNS	COR	COE	AFE
PCLC	-				
RNS	0.344	-			
COR	0.367	0.379	-		
COE	0.473	0.372	0.427	-	
AFE	0.459	0.383	0.445	0.562	-

PCLC, perceived cooperative learning climate in physical education; RNS, relatedness need satisfaction in physical education; COR, cognitive reappraisal; COE, cognitive empathy; AFE, affective empathy. Values below 0.85 indicate satisfactory discriminant validity under a conservative criterion; values below 0.90 indicate acceptable discriminant validity under a more lenient criterion. All HTMT values in the present study were below 0.85.

### 4.4 Multiple regression analysis

Hierarchical multiple regression analyses were conducted as supplementary covariate-adjusted analyses, with cognitive empathy (COE) and affective empathy (AFE) as dependent variables. Demographic variables (gender, grade, residential area, only-child status, and parental education level) were entered as control variables in the first step, followed by PCLC in the second step, and RNS and COR in the final step. Standardized regression coefficients (β) are reported in Table 6.

**Table 6:** Supplementary hierarchical multiple regression analyses for cognitive empathy and affective empathy (*N* = 2,173).

Variables	Model 1 (COE)		Model 2 (AFE)	
	β	SE	β	SE
Control variables				
Gender	0.037^*^	0.032	0.066^***^	0.033
Grade	−0.035	0.019	0.025	0.020
Residential area	−0.016	0.019	0.006	0.020
Only-child status	−0.030	0.031	0.018	0.033
Parental education (Highest level)	0.106^***^	0.014	0.077^**^	0.015
Independent variable				
PCLC	0.270^***^	0.024	0.253^***^	0.025
Indirect-pathway variables				
RNS	0.156^***^	0.018	0.172^***^	0.018
COR	0.229^***^	0.019	0.252^***^	0.020
Model summary				
*R* ^2^	0.279		0.285	
Adjusted *R*^2^	0.277		0.283	
*F*	104.787^***^		108.056^***^	

Standardized coefficients (β) are reported. PCLC, Perceived cooperative learning climate in physical education; RNS, Relatedness need satisfaction in physical education; COR, cognitive reappraisal; COE, cognitive empathy; AFE, affective empathy. ^*^ *p* < 0.05, ^**^ *p* < 0.01, ^***^ *p* < 0.001. Demographic coefficients were included for covariate adjustment and were not interpreted as focal estimates.

For COE (Model 1), parental education was positively associated with COE (β = 0.106, *p* < 0.001), whereas gender, grade, residential area, and only-child status were not consistently associated with COE. After demographic variables were entered, PCLC was positively associated with COE (β = 0.270, *p* < 0.001). When RNS and COR were entered, both variables were positively associated with COE (RNS: β = 0.156, *p* < 0.001; COR: β = 0.229, *p* < 0.001). The overall model explained 27.9% of the variance in COE (*R*^2^ = 0.279, Adjusted *R*^2^ = 0.277), *F* = 104.787, *p* < 0.001.

For AFE (Model 2), gender (β = 0.066, *p* < 0.001) and parental education (β = 0.077, *p* < 0.01) were positively associated with AFE, whereas grade, residential area, and only-child status were not consistently associated with AFE. PCLC remained positively associated with AFE (β = 0.253, *p* < 0.001). After RNS and COR were entered, both variables were positively associated with AFE (RNS: β = 0.172, *p* < 0.001; COR: β = 0.252, *p* < 0.001). The model accounted for 28.5% of the variance in AFE (*R*^2^ = 0.285, Adjusted *R*^2^ = 0.283), *F* = 108.056, *p* < 0.001.

Overall, the regression results indicated that PCLC, RNS, and COR were positively associated with both COE and AFE after demographic variables were entered. These analyses provided supplementary covariate-adjusted evidence for the associations among the focal variables.

### 4.5 Structural equation modeling analysis

The hypothesized structural model was estimated in AMOS 26.0 on the basis of the previously supported five-factor measurement model. Specifically, the same latent measurement structure for PCLC, RNS, COR, COE, and AFE was retained, and directional structural paths were then specified among the latent variables in accordance with the hypothesized model. The structural model included paths from PCLC to RNS, COR, COE, and AFE; paths from RNS to COR, COE, and AFE; and paths from COR to COE and AFE. COE and AFE were specified as correlated endogenous variables. All reported coefficients are standardized estimates. The SEM was estimated without demographic covariates; therefore, the reported structural paths represent unadjusted latent-variable estimates.

**Figure 2:**
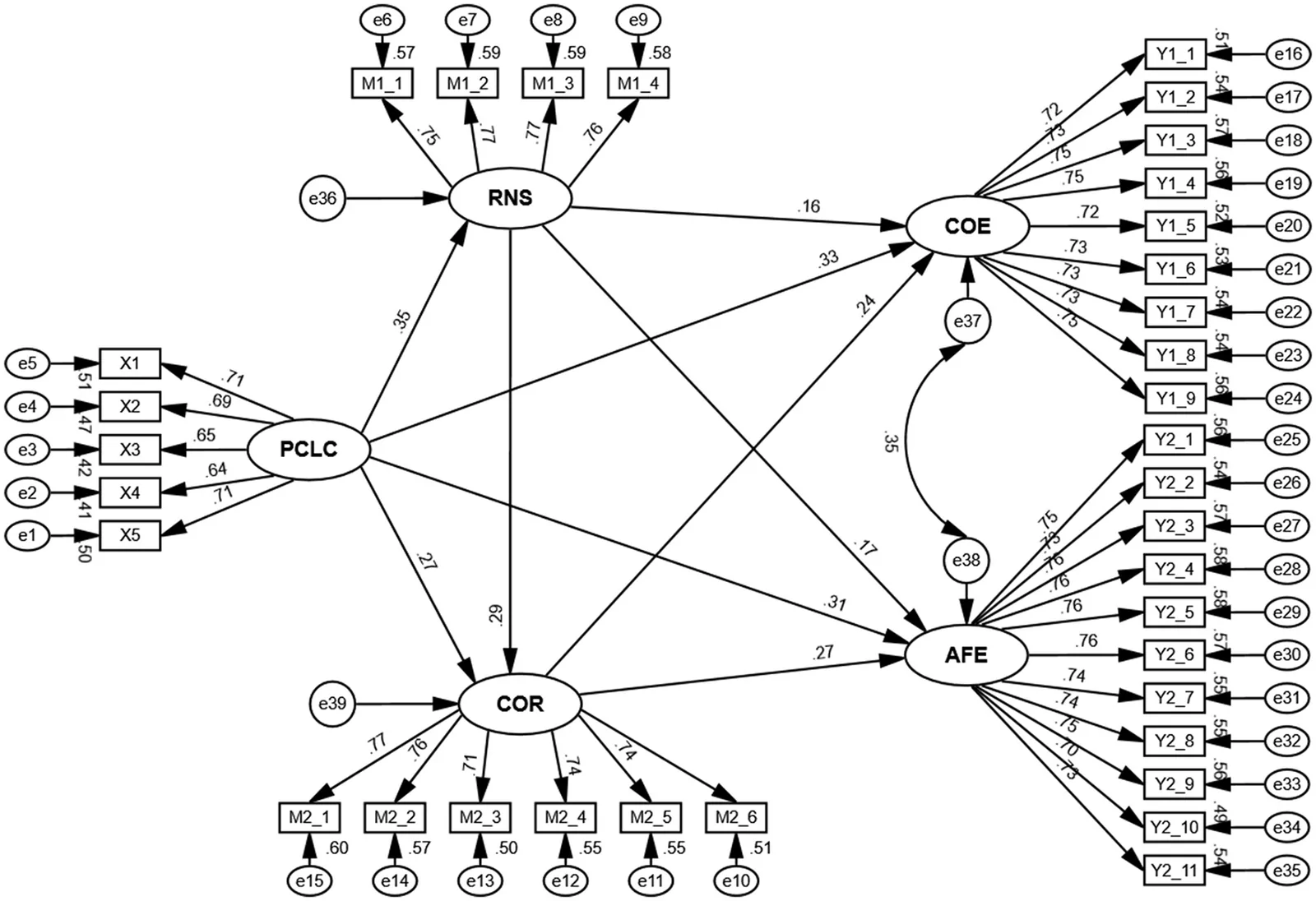
Structural equation model linking cooperative learning climate, relatedness need satisfaction, cognitive reappraisal, and empathy. Standardized path coefficients are presented. The SEM did not include demographic covariates; coefficients represent unadjusted estimates. All displayed paths are statistically significant at *p* < 0.05. PCLC, Perceived cooperative learning climate in physical education; RNS, Relatedness need satisfaction in physical education; COR, Cognitive reappraisal; COE, Cognitive empathy; AFE, Affective empathy.

The unadjusted structural model demonstrated excellent fit to the data, χ^2^/df = 2.600, CFI = 0.977, TLI = 0.975, SRMR = 0.027, and RMSEA = 0.027, 90% CI [0.025, 0.029]. As shown in Figure 2, PCLC was positively associated with RNS (β = 0.35, *p* < 0.001) and COR (β = 0.27, *p* < 0.001). PCLC was also directly associated with both COE (β = 0.33, *p* < 0.001) and AFE (β = 0.31, *p* < 0.001). RNS was positively associated with COR (β = 0.29, *p* < 0.001), as well as with COE (β = 0.16, *p* < 0.001) and AFE (β = 0.17, *p* < 0.001). In addition, COR was positively associated with COE (β = 0.24, *p* < 0.001) and AFE (β = 0.27, *p* < 0.001). All specified structural paths were statistically significant.

### 4.6 Bootstrapping test of indirect effects

To examine the significance of indirect effects, a bias-corrected bootstrap procedure with 5,000 resamples was conducted. Indirect effects were considered statistically significant when the 95% confidence interval (CI) did not include zero. The results for the direct, indirect, and total effects are presented in Table 7. These estimates were derived from the unadjusted SEM.

**Table 7:** Bootstrapping results for direct and indirect effects (*N* = 2,173).

Path	Estimate	Boot SE	Boot LLCI	Boot ULCI
Direct effect				
PCLC → COE	0.334^***^	0.025	0.284	0.382
PCLC → AFE	0.308^***^	0.025	0.258	0.357
Indirect effects				
PCLC → RNS→ COE	0.057^***^	0.009	0.039	0.076
PCLC → COR → COE	0.066^***^	0.009	0.051	0.085
PCLC → RNS→ COR → COE	0.024^***^	0.004	0.018	0.032
PCLC → RNS→ AFE	0.060^***^	0.009	0.044	0.080
PCLC → COR → AFE	0.073^***^	0.009	0.056	0.093
PCLC → RNS→ COR → AFE	0.027^***^	0.004	0.020	0.035
Total effect				
PCLC → COE	0.482^***^	0.021	0.439	0.520
PCLC → AFE	0.468^***^	0.021	0.426	0.508

PCLC, perceived cooperative learning climate in physical education; RNS, relatedness need satisfaction in physical education; COR, cognitive reappraisal; COE, cognitive empathy; AFE, affective empathy. Boot LLCI, the lower bound of the 95% confidence interval. Boot ULCI, the upper limit of the 95% confidence interval (bias-corrected percentile bootstrap method). Bootstrap sample size = 5,000. ^***^ *p* < 0.001.

For cognitive empathy (COE), the direct effect of PCLC was significant (β = 0.334, Boot SE = 0.025, 95% CI [0.284, 0.382]). Three specific indirect effects were also significant: PCLC → RNS→ COE (β = 0.057, Boot SE = 0.009, 95% CI [0.039, 0.076]), PCLC → COR → COE (β = 0.066, Boot SE = 0.009, 95% CI [0.051, 0.085]), and PCLC → RNS→ COR → COE (β = 0.024, Boot SE = 0.004, 95% CI [0.018, 0.032]). The total effect of PCLC on COE was β = 0.482, Boot SE = 0.021, 95% CI [0.439, 0.520].

For affective empathy (AFE), the direct effect of PCLC was significant (β = 0.308, Boot SE = 0.025, 95% CI [0.258, 0.357]). The specific indirect effects through RNS (β = 0.060, Boot SE = 0.009, 95% CI [0.044, 0.080]), through COR (β = 0.073, Boot SE = 0.009, 95% CI [0.056, 0.093]), and through RNS followed by COR (β = 0.027, Boot SE = 0.004, 95% CI [0.020, 0.035]) were significant. The total effect of PCLC on AFE was β = 0.468, Boot SE = 0.021, 95% CI [0.426, 0.508].

Across both outcomes, all specific indirect effects were statistically significant, and all bootstrapped confidence intervals excluded zero. Given the large sample size, interpretation emphasized standardized effect magnitudes. Specific indirect effects ranged from β = 0.024 to β = 0.073. The total indirect effects were β = 0.147 for COE and β = 0.160 for AFE, accounting for 30.5% and 34.2% of the corresponding total effects, respectively.

## 5 Discussion

### 5.1 Main findings and effect sizes

This study examined whether perceived cooperative learning climate in physical education was associated with cognitive empathy and affective empathy among Chinese upper elementary students, and whether relatedness need satisfaction and cognitive reappraisal served as relational and regulatory indirect pathways. The findings suggest that students who perceived a more cooperative learning climate in PE also reported higher cognitive and affective empathy. RNS and COR were positively associated with both empathy components, and the specific indirect effects through RNS, through COR, and through the sequence RNS→ COR were statistically significant for both outcomes.

Given the large sample size, the interpretation should focus not only on statistical significance but also on effect magnitude. The direct effects of PCLC on COE and AFE were larger than the specific indirect effects, with standardized estimates of β = 0.334 and β = 0.308, respectively. The specific indirect effects were smaller, ranging from β = 0.024 to β = 0.073. The total indirect effects were β = 0.147 for COE and β = 0.160 for AFE, accounting for 30.5% and 34.2% of the corresponding total effects. These values suggest that relational and regulatory pathways were meaningful within the hypothesized model, but their magnitude should be interpreted as modest. The SEM estimates were unadjusted for demographic covariates, whereas supplementary hierarchical regressions indicated that the focal associations remained positive after demographic variables were entered.

The similar pattern observed for COE and AFE also deserves attention. Cognitive empathy and affective empathy were empirically distinguishable in the measurement model, yet both were associated with PCLC, RNS, and COR in broadly comparable ways. This pattern suggests that cooperative PE climates may be relevant to multiple facets of empathic functioning, including both perspective-oriented understanding and emotional responsiveness.

### 5.2 Relational and regulatory interpretation

The positive association between PCLC and both empathy components is consistent with the view that cooperative learning in PE may provide a meaningful social context for empathy-related development. Cooperative learning structures emphasize positive interdependence, promotive interaction, shared responsibility, and group reflection. These features may make peers' perspectives, emotions, and reactions more visible during class activity (Železnik Mežan et al., 2025). In PE, students often coordinate movement with classmates, perform in front of others, and respond to success, failure, and feedback in real time. Such conditions create repeated opportunities to notice how others feel, adjust one's behavior, and interpret classmates' reactions (Kokkonen et al., 2020). The present findings therefore extend existing perspectives on cooperative learning in physical education by linking classroom interaction structure with socio-emotional correlates rather than only with motivational or performance-related outcomes (Boke et al., 2025; Rivera-Pérez et al., 2021).

RNS provides one possible relational explanation for these associations. From a self-determination perspective, relatedness refers to students' sense of belonging, acceptance, and interpersonal connection. In PE, where peer interaction is frequent and visible, this form of relational security may be especially salient (Xiang et al., 2017). Students who feel accepted in PE may have more attentional and emotional space to engage with classmates' experiences rather than focusing primarily on self-protection or social threat. This interpretation is consistent with prior work linking relatedness, interpersonal support, and socio-emotional functioning (Cox et al., 2009; Legg et al., 2025; Slemp et al., 2024). In the present model, RNS was positively associated with both COE and AFE, suggesting that students' relational comfort in PE may be linked with both understanding others' perspectives and responding emotionally to others' experiences.

COR provides a complementary regulatory explanation. Cognitive reappraisal involves changing the meaning of a situation so that one's emotional response becomes more manageable (Feldon, 2024). PE lessons frequently include emotionally salient moments, such as public mistakes, disagreement, peer feedback, competition, and coordination difficulties. Students who more often reinterpret such situations constructively may be better able to remain open to classmates' perspectives and emotions rather than becoming dominated by self-focused distress. This interpretation aligns with research linking emotion regulation with social cognition and empathy-related processes (Lauren et al., 2016; Morawetz et al., 2022; Xu et al., 2023). In the present model, COR was positively associated with both empathy components, and the specific indirect effects through COR were slightly larger than those through RNS.

The sequential pathway through RNS followed by COR adds a further layer to this interpretation. Although the sequential indirect effects were smaller than the direct effects and the two single-mediator indirect effects, they appeared for both empathy outcomes. This pattern is consistent with a relational–regulatory configuration in which cooperative PE climates are linked with stronger relatedness, stronger relatedness is linked with greater cognitive reappraisal, and both processes are associated with empathy. Students who feel more accepted and connected in PE may appraise emotionally demanding peer situations as less threatening, making constructive reinterpretation more feasible. This interpretation is compatible with work suggesting that interpersonal security and psychological need satisfaction may be linked with more adaptive emotion regulation (Robazza et al., 2023; Xiang et al., 2017). At the same time, this sequence should not be treated as evidence of a fixed developmental mechanism. The cross-sectional design supports a theoretically coherent statistical pattern, not temporal or causal ordering.

### 5.3 Theoretical and practical implications

The findings contribute to the literature in three main ways. The study extends research on cooperative learning in PE by connecting perceived cooperative climate with empathy-related correlates in late childhood. Much of the PE literature has emphasized motivation, engagement, or performance outcomes. The present findings suggest that cooperative instructional climates may also be relevant to students' socio-emotional functioning.

The study also brings together self-determination theory and emotion regulation perspectives within a common framework. By examining RNS and COR alongside PCLC, the model links students' relational experience in PE with their tendency to reinterpret emotionally charged situations. This integration is consistent with the idea that motivational and regulatory perspectives can be complementary when examining socio-emotional processes in educational settings (Neufeld, 2025). In addition, treating COE and AFE as distinct but correlated outcomes supports a more differentiated account of empathy, rather than treating empathy as a single undifferentiated disposition.

The practical implications should be interpreted cautiously because the study did not test an intervention. Still, the findings point to several promising directions for PE practice and future intervention design. PE teachers may consider structuring group activities around positive interdependence, shared responsibility, and guided peer interaction. Teacher perspective taking may also support autonomy-supportive implementation of these practices (Reeve and Cheon, 2024). Attention to grouping practices, peer dialogue, and classroom norms may also help support students' sense of belonging and interpersonal comfort. For emotion regulation, brief debriefing prompts, opportunities to reframe setbacks, and guided reflection after challenging group activities may be worth exploring, especially in lessons involving public mistakes, disagreement, or competition. These suggestions should be regarded as hypothesis-generating rather than prescriptive.

### 5.4 Limitations and future directions

Several limitations should be considered. The cross-sectional design limits conclusions about temporal ordering. Although the hypothesized model was theoretically grounded, the direct and indirect effects should not be interpreted as evidence of causal or developmental sequencing. Longitudinal, cross-lagged, and intervention-based designs are needed to examine how perceived cooperative learning climate, RNS, COR, and empathy may become linked over time.

All focal variables were assessed through student self-report, which may have inflated the observed associations because instructional climate, relational experience, emotion regulation, and empathy were reported by the same respondents. Although procedural remedies and statistical checks were used to assess common method variance, shared source effects and subjective bias cannot be ruled out. Future research would benefit from multi-informant and multi-method designs, including teacher ratings, peer nominations, behavioral observations, or brief social desirability measures.

The SEM indirect effects were estimated without demographic covariates. Supplementary hierarchical regressions indicated that the focal associations remained positive after demographic variables were entered, but the reported SEM direct and indirect effects should be interpreted as unadjusted latent-variable estimates. Future studies could estimate covariate-adjusted SEMs or compare adjusted and unadjusted models as robustness checks.

Several additional methodological considerations remain. The 5-point Likert responses were treated as approximately continuous and analyzed using maximum likelihood estimation in AMOS. This practice is common in large-sample research, but future studies could use estimators designed for ordered-categorical indicators or conduct robustness checks with alternative estimation methods. Although class-level clustering appeared limited, the nested sampling structure should still be considered in future work. Measurement invariance across gender and grade was also not formally tested; future studies should examine configural, metric, and scalar invariance before drawing stronger subgroup comparisons.

Finally, the sample was drawn from primary schools in Chengdu, China. Schools were recruited partly on the basis of access and willingness to participate, although students were randomly selected within participating classes. This sampling design limits the generalizability of the findings to broader populations. Cultural norms surrounding cooperation, emotional expression, peer interaction, and classroom authority may shape how cooperative learning climates are perceived and how empathy is expressed in PE. Replication across different regions, school systems, and cultural contexts would strengthen external validity. Future research may also incorporate additional contextual factors, such as instructional style, teacher behaviors, class size, and school-level characteristics.

## 6 Conclusion

This study provides cross-sectional evidence that perceived cooperative learning climate in PE is positively associated with cognitive and affective empathy among Chinese upper elementary students. RNS and COR may represent relational and regulatory indirect pathways within the hypothesized statistical model, although the specific indirect effects were modest and the SEM estimates were unadjusted for demographic covariates. By integrating cooperative instructional climate, relatedness need satisfaction, cognitive reappraisal, and two components of empathy, the study offers a more differentiated account of socio-emotional functioning in PE. The findings suggest the potential value of structured cooperative environments for empathy-related dispositions in late childhood, while also underscoring the need for longitudinal, multi-informant, and intervention-oriented research to test temporal and causal relations.

## Data Availability

The original contributions presented in the study are included in the article/Supplementary material, further inquiries can be directed to the corresponding author.
